# Using Accelerometer Data to Tune the Parameters of an Extended Kalman Filter for Optical Motion Capture: Preliminary Application to Gait Analysis

**DOI:** 10.3390/s21020427

**Published:** 2021-01-09

**Authors:** Javier Cuadrado, Florian Michaud, Urbano Lugrís, Manuel Pérez Soto

**Affiliations:** Laboratory of Mechanical Engineering, University of La Coruña, 15403 Ferrol, Spain; javier.cuadrado@udc.es (J.C.); florian.michaud@udc.es (F.M.); manuel.perez.soto@udc.es (M.P.S.)

**Keywords:** Kalman filter, motion capture, gait analysis, inertial sensor

## Abstract

Optical motion capture is currently the most popular method for acquiring motion data in biomechanical applications. However, it presents a number of problems that make the process difficult and inefficient, such as marker occlusions and unwanted reflections. In addition, the obtained trajectories must be numerically differentiated twice in time in order to get the accelerations. Since the trajectories are normally noisy, they need to be filtered first, and the selection of the optimal amount of filtering is not trivial. In this work, an extended Kalman filter (EKF) that manages marker occlusions and undesired reflections in a robust way is presented. A preliminary test with inertial measurement units (IMUs) is carried out to determine their local reference frames. Then, the gait analysis of a healthy subject is performed using optical markers and IMUs simultaneously. The filtering parameters used in the optical motion capture process are tuned in order to achieve good correlation between the obtained accelerations and those measured by the IMUs. The results show that the EKF provides a robust and efficient method for optical system-based motion analysis, and that the availability of accelerations measured by inertial sensors can be very helpful for the adjustment of the filters.

## 1. Introduction

Human motion capture during gait provides a way to understand the principles of the natural mode of locomotion of the human being. Technological advances have changed its practice and improved its accuracy along history [1,2]. Recent developments in microelectromechanical systems (MEMS) have caused a renewed interest in the use of inertial measurement units (IMUs) to perform three-dimensional (3D) human movement reconstruction [3,4,5,6,7,8,9,10]. However, getting orientation from IMUs presents accuracy and consistency issues [11,12,13,14,15,16], especially in the presence of environmental ferromagnetic disturbances or when measuring fast complex movements over long periods of time [17]. This is why, although the performance of inertial sensors has improved in the last decade, optical motion capture remains as the preferred method to perform precise biomechanical studies. In fact, as pointed out in [18,19], IMU-based methods for motion capture and reconstruction are usually validated against optical methods, which remain as the golden standard reference. The problem with optical motion capture systems is that it is very difficult to ensure that all markers are visible to the cameras all the time and, moreover, other reflective objects present in the capture zone can be incorrectly identified as markers. In general, obtaining the skeletal motion involves some manual post-processing of the captured data, so the technique is not straightforward [20,21]. This problem can be overcome by using an extended Kalman filter (EKF) [22], as will be described later in this paper.

The typically high-frequency noise harmonics present in the recorded marker trajectories are hardly perceptible at displacement level. However, after numerical differentiation, the amplitude of each harmonic increases with its harmonic number; for velocities, it increases linearly, and, for accelerations, the increase is proportional to the square of the harmonic number [2]. For this reason, filtering is required when trying to obtain velocities and accelerations by numerically differentiating position data. The problem here lies on the choice of the cutoff frequency of the filter, since it is difficult to achieve a value that filters out most of the noise, without also removing relevant motion information [23,24,25,26,27]. From what has been said, it is deduced that different filtering quality is demanded for the animation of virtual characters, where only configuration-level information is needed, and for biomechanical analysis including dynamics, where velocity- and acceleration-level information is also required.

Numerous filtering approaches can be found in the literature [2,23,28,29]. While the Butterworth filter has generally been preferred, because impulsive-type inputs are rarely seen in human movement data [28,30,31], recent studies have applied the Kalman filter [32] (most commonly used in the literature for inertial sensors), thus improving the accuracy of estimated joint kinematics and computed orientation data [22,33,34]. However, beyond the choice of a filtering algorithm, the main problem remains in the tuning of its parameters. In almost all the filtering studies for gait analysis, the smoothing level is based on the author’s decision of how much noise is acceptable. A common criterion is to establish an error threshold at position level, and then setting the cutoff frequency accordingly [22,23]. Regardless of the method used, there is no way of assessing the accuracy of the obtained accelerations by relying on position data alone. In order to provide an objective filter-tuning procedure, this work proposes to compare the filtered accelerations from the optical capture with their experimental measurement from the inertial sensors in the case of gait. In [24], a similar procedure was applied in the case of jumping, but it was not successful due to the overshoot provided by the accelerometers in their horizontal measurements.

IMUs are capable of estimating their own orientation within an Earth-fixed frame by using sensor fusion algorithms, such as Madgwick’s algorithm [35] or the EKF [36]. These algorithms provide an estimate of the orientation by combining the information from the triaxial accelerometers, gyroscopes and magnetometers present in the IMU. Because IMUs show limitations to give an accurate orientation [13] (closely related to sensor calibration, magnetometer sensitivity, and presence of accelerations other than gravity) and, moreover, their Earth-based global frame will in general differ from that of the optical motion capture system, a preliminary test was performed with nine IMUs. This test was carried out in order to assess the instrumental errors associated, select the most accurate units, and determine the global frame offset corresponding to each one [11]. Second, the gait analysis of a healthy subject was conducted. The motion was recorded by both the optical and the inertial techniques, using the seven most accurate IMUs among the nine previously tested. The human motion was then reconstructed by using both the classic Vaughan’s method [37], which does not impose the kinematic constraints and is similar to those proposed in [19] and the EKF introduced in this paper, which allows automatic marker labeling, is robust to short marker occlusions and imposes kinematic constraints, even in real time, so the local accelerations measured by the IMUs could be used to tune the filters applied to the optical motion capture data.

The remaining of the paper is organized as follows. Section 2 describes the two experiments carried out, sensor test and gait analysis. It points out the errors that may be incurred by inertial sensors, and proposes a way to minimize their influence. Then, it explains the two motion reconstruction methods applied and compared in this work, with a detailed description of the EKF, and shows the procedure to obtain the accelerations of the IMU attachment points from the optical system-based analysis, so that they can be compared with the accelerations measured by the IMUs. Section 3 presents the results of both the preliminary test and the gait analysis, showing the errors of the inertial sensors in orientations and accelerations, and the effect of the filter parameters adopted for the motion reconstructions methods in the accuracy of the accelerations obtained from the optical system recordings. Finally, Section 4 discusses the results and points out the limitations of the study, while Section 5 draws the conclusions of the work.

## 2. Materials and Methods

### 2.1. Preliminary Test

IMUs provide the measured accelerations expressed in their local reference frames, while the optical motion capture system provides marker positions within its own fixed reference frame. Therefore, in order to establish a comparison between accelerations coming from both techniques, it is necessary to express them in the same reference frame, and this implies obtaining the transformation matrix between each IMU and the fixed frame used by the optical motion capture system.

As mentioned above, an IMU can use sensor fusion algorithms to estimate its orientation within an Earth-fixed frame. This Earth-fixed frame is usually defined as NED (North-East-Down) or NWU (North-West-Up), and will be probably rotated with respect to the reference frame used by the motion capture lab. Therefore, the first step is to determine the offset between both reference frames, for which two methods can be applied: (i) the first option is to carry out a preliminary IMU calibration process, as the spot check proposed in [11]; (ii) the second alternative is to attach three markers to each IMU, so the local frames can be obtained directly from the optical motion capture system [38]. Since the second method requires a large number of markers, thus making the motion capture process more involved and error-prone, the calibration approach has been chosen in this work.

#### 2.1.1. Experimental Data Collection

**Figure 1:**
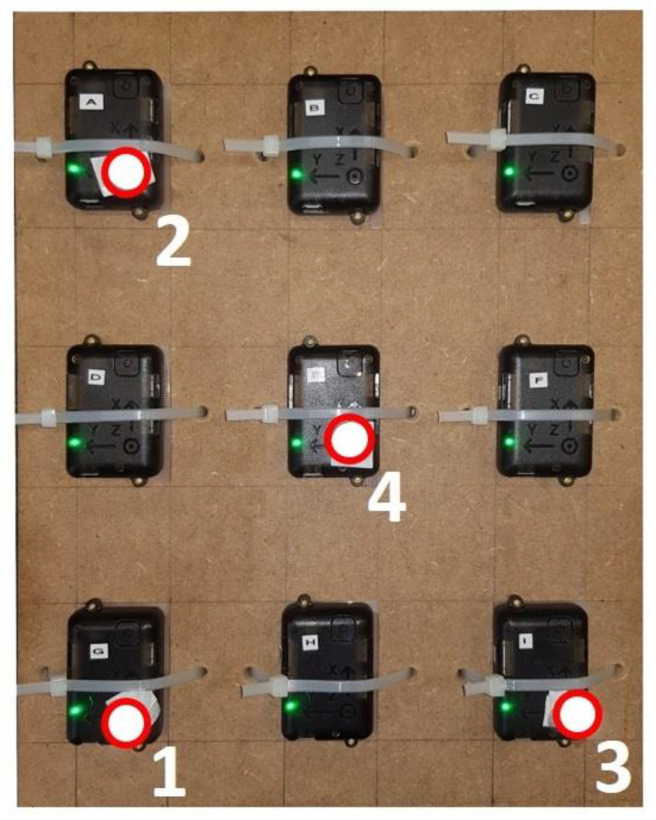
Calibration setup composed by nine IMUs and four markers on a rigid plate.

Nine IMUs (STT-IWS, STT Systems, San Sebastián, Spain) sampling at 100 Hz were fixed on a flat rigid wooden plate (with no ferromagnetic disturbances), equally spaced and accurately aligned to each other. Four reflective markers were also attached to four of the sensors, as illustrated in Figure 1.

The optical motion capture system was formed by 18 infrared cameras (OptiTrack FLEX 3, Natural Point, Corvallis, OR, USA), also sampling at 100 Hz. Starting with the plate on the floor, where it was kept for 5 s, it was manually moved around for 30 s and, finally, put again in the original place during 5 s. Data from both the IMU set and the optical system were recorded and the plate orientation during the motion was obtained from: (i) each IMU, based on gravity, magnetic North and gyroscope integration within the commercial software iSen provided by the manufacturer; (ii) the optical system, by rigid-body motion reconstruction based on the trajectories of the reflective markers 1, 2 and 3.

#### 2.1.2. Sensor Orientation and Geomagnetic Frame of Reference

**Figure 2:**
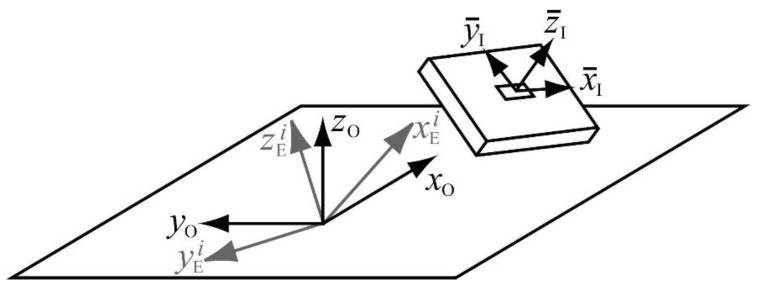
The three reference frames involved in the calibration: fixed global reference frame of the optical system (subscript O); Earth-fixed global reference frame of each IMU (in grey, subscript E and superscript i); moving local reference frame of all the IMUs and the wooden plate (subscript I).

Figure 2 shows the three reference frames involved in the problem. The first reference frame is the global reference frame of the motion capture lab, obtained after calibration of the optical system, and it is noted with subscript O (after optical). This reference frame is fixed and common for all IMUs. The second reference frame is the global, Earth-fixed reference frame of each inertial sensor, and it is noted with subscript E (after Earth-fixed), and superscript *i* denoting the IMU number. Although this frame should be the same for all the IMUs, their inherent errors in determining gravity and magnetic North directions lead to discrepancies among sensors. The third reference frame is the local reference frame of each inertial sensor, and it is noted with subscript I (after inertial). In the calibration setup, the local reference frame is the same for all the IMUs, and it coincides with the local reference frame of the wooden plate. Note that the axes of this reference frame have a bar on them, meaning that they are moving axes, rigidly attached to the wooden plate.

If $R_{\mathrm{OI}}$ is the variable rotation matrix that transforms the components of a vector expressed in the reference frame I into the components of the same vector expressed in the reference frame O, $R_{\mathrm{OE}}^{i}$ is the constant rotation matrix that does the same between frames E and O for the inertial sensor i, and $R_{\mathrm{EI}}^{i}$ is the variable rotation matrix that makes the same between frames I and E for that sensor, the following relation can be stated at any instant of the plate motion:

(1) $$R_{\mathrm{OI}}=R_{\mathrm{OE}}^{i}R_{\mathrm{EI}}^{i}$$

At any time point, the trajectories of the markers measured by the optical system provide $R_{\mathrm{OI}}$, while the sensor fusion algorithm from the ith IMU provides $R_{\mathrm{EI}}^{i}$. Therefore $R_{\mathrm{OE}}^{i}$ can be derived from Equation (1) as,

(2) $$R_{\mathrm{OE}}^{i}=R_{\mathrm{OI}}R_{\mathrm{EI}}^{i\;T}$$

Each $R_{\mathrm{OE}}^{i}$ matrix must be constant, since it represents a rotation between two fixed frames. However, if it is calculated for all the time points of the recorded motion, the obtained values will not be completely constant, due to sensor and estimation errors. In order to find a unique matrix for each IMU, an average rotation matrix is calculated and taken as its effective $R_{\mathrm{OE}}^{i}$. Since rotation matrices are orthogonal, care must be taken when averaging them, so that orthogonality is preserved. The method followed here consisted of extracting the roll, pitch and yaw angles from each rotation matrix at every time point, averaging them, and using these values to build back the corresponding effective rotation matrix. This calibration procedure to get the $R_{\mathrm{OE}}^{i}$ matrices yields different results for different days due to magnetic changes, so it should be ideally performed right before using the IMUs.

Once the IMUs were properly calibrated, the orientation error provided by each of them along the motion of the wooden plate was obtained. For each time point, the trajectories of the markers measured by the optical system provided matrix $R_{\mathrm{OI}}$, while data from ith IMU provided $R_{\mathrm{EI}}^{i}$. Since the constant matrix $R_{\mathrm{OE}}^{i}$ had been obtained for each IMU in the calibration process described before, it can be written from Equation (1),

(3) $$R_{\mathrm{OI}}^{i}=R_{\mathrm{OE}}^{i}R_{\mathrm{EI}}^{i}$$

where $R_{\mathrm{OI}}^{i}$ is the rotation matrix between frames I and O provided by the ith inertial sensor. Ideal IMUs would provide the same matrix $R_{\mathrm{OI}}^{i}$ for all the sensors, and it would be coincident with matrix $R_{\mathrm{OI}}$ provided by the optical system. However, due to errors in the IMUs, such matrices differ, and the orientation error committed by each IMU at each time point can then be obtained by calculating the roll, pitch and yaw angles of $R_{\mathrm{OI}}^{i}$, and comparing them with the roll, pitch and yaw angles of $R_{\mathrm{OI}}$, taken as reference. This was done for the nine IMUs, the results being shown in Section 3.

Once the method to obtain the orientation error of each inertial sensor has been described, the objective of comparing the accelerations provided by the optical and the inertial systems is addressed. The optical system provides the trajectories of the markers, based on which the position history of any point of the body can be obtained through a motion reconstruction method. Then, double differentiation of the position history yields velocity and acceleration histories of the point considered. Positions, velocities and accelerations are expressed in the global reference frame of the motion capture lab (previously denoted by O). On the other hand, IMU accelerometers measure a combination of the gravitational and translational accelerations. As reported by Woodman [21], it is necessary to have very accurate rotation sensors in inertial navigation systems, because knowing the precise orientation of the body allows to properly subtract the gravitational acceleration from the measurement, in order to find the translational acceleration. Each IMU provides its acceleration expressed in its local reference frame (previously denoted by I). Therefore, to compare accelerations obtained through the optical and inertial techniques, it is necessary to express them in the same reference frame and to take into account the gravity constant, which is present in the inertial case.

To highlight all the mentioned issues, the acceleration of point 4 in Figure 1 was obtained in three different ways. First, since point 4 had a marker on it, the marker trajectory was filtered by means of a 8 Hz forward-backward 2nd order Butterworth filter, then it was differentiated twice with respect to time, and the gravity constant (9.81 m/s^2^) was added to the vertical component of the resulting acceleration; the presence of the marker attached at the point made it unnecessary the use of any motion reconstruction method, thus eliminating a source of error for the optical system. Second, the acceleration provided by the IMU at point 4 was expressed in frame O by multiplying it by matrix $R_{\mathrm{OI}}^{5}$ (orientation provided by the IMU #5, attached to that point). Third, the acceleration provided by the IMU at point 4 was expressed in frame O by multiplying it by matrix $R_{\mathrm{OI}}$ (orientation provided by the optical system after the mentioned filtering of the marker trajectory). The resulting accelerations and their comparison are shown in Section 3.

### 2.2. Gait Analysis

#### 2.2.1. Experimental Data Collection

**Figure 3:**
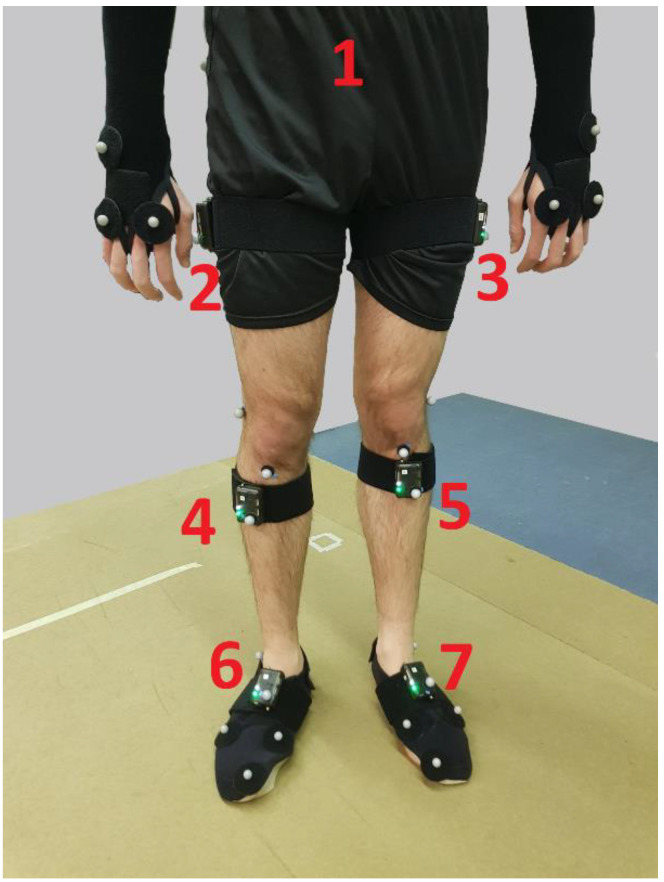
Markers and IMUs (red numbers) attached to the subject’s body for gait analysis.

A healthy adult male, 24 years old, 70 kg, and 175 cm, performed a complete gait cycle. Both 36 reflective markers in all his body segments for optical motion capture (same equipment as that described in Section 2.1) and 7 IMUs (the best seven among the nine tested in the preliminary test) at pelvis, thighs, shanks and feet for inertial motion capture were attached to the subject’s body, as can be seen in Figure 3. One additional marker was attached to each IMU so as to determine its local position within the corresponding segment during a static pose recording.

#### 2.2.2. Skeletal Model and Kinematics

**Figure 4:**
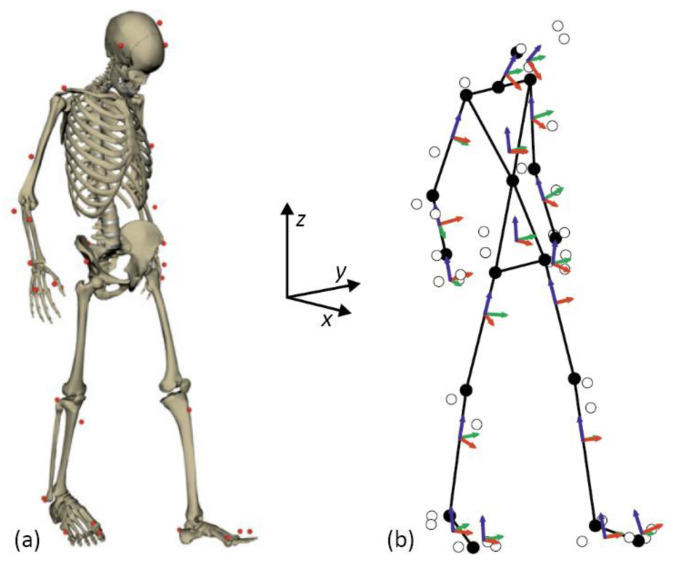
3D human model: (**a**) graphical output; (**b**) multibody model showing the segments: joints (black dots), and marker locations (white dots).

The human body is modeled as a three-dimensional multibody system formed by rigid bodies, as shown in Figure 4. The model consists of 18 anatomical segments [39]: two hind feet, two forefeet, two shanks, two thighs, pelvis, torso, neck, head, two arms, two forearms, and two hands. The segments are linked by ideal spherical joints (black dots in Figure 4b), thus defining a model with 57 degrees of freedom (DOF). The axes of the global reference frame are defined as follows: *x*-axis in the antero-posterior direction, *y*-axis in the medio-lateral direction, and *z*-axis in the vertical direction.

#### 2.2.3. Motion Reconstruction from Motion Capture Data

Optical motion capture records the motion of entities (markers) that are external to the body, and the objective is to use the marker data to determine the positions and orientations of the body segments. The traditional approach for accomplishing this is to use the method described by Vaughan [37]: (i) select three non-collinear entities, which can be either markers or already located joints, within each body segment; (ii) define an orthogonal reference frame for the corresponding segment, based on the three selected entities; (iii) use correlation equations, based on archived anthropometric data and body measurements, to estimate the position and orientation of the body segment. When applying this method, marker trajectories are previously filtered with a low-pass filter (forward-backward 2nd order Butterworth filter), whose cutoff frequency must be selected by the analyst.

Another commonly used approach is to solve a weighted optimization problem, in order to fit the skeletal model to the measured markers, as done in the OpenSim software [40]. The fitting is carried out in two steps. First, a reference skeletal model, with virtual markers fixed to the anatomical points, is scaled in order to match the markers from a static capture, taken in a reference pose. Then, a second optimization problem finds the positions and orientations of the scaled body segments that best track the motion capture data. This last optimization uses an independent set of positions and orientations as design variables, which can be filtered and differentiated afterwards to find velocities and accelerations.

These methods present important drawbacks. In the first one, the local frames are obtained directly from the markers, which are not rigidly attached to the bones, so the obtained skeletal motion is not consistent with the rigid body constraints, i.e., the distances between joints do not remain constant. This can be addressed by enforcing kinematic consistency in a post-processing stage [41]. Another problem, common to both methods, is that they require clean capture data: marker trajectories must not contain any gaps, and the markers need to be properly labeled at every time step, something that is not always guaranteed by the motion capture system. The procedure used for fixing this problem usually involves some manual gap filling and marker labeling [20] and, in some cases of severe marker loss, it is not even possible to salvage a take. The main consequence of these drawbacks is the impossibility of knowing if a motion capture take has been successful until all post-processing has been carried out.

#### 2.2.4. Extended Kalman Filter for Motion Reconstruction

In order to overcome these drawbacks, a motion capture algorithm based on the extended Kalman filter has been developed. The filter uses a purely kinematic model for the plant, while the markers act as position sensors. The kinematic model mostly coincides with that described in Figure 4 but, in order to avoid the need for additional markers, the spherical joint at the base of the neck has been substituted by a universal joint, and metacarpophalangeal joints are modeled here as revolute pairs. Therefore, the kinematic model used in the Kalman filter has 52 degrees of freedom instead of 57. Since the Kalman filter requires using independent state variables, the position of the model must be defined by a set of independent coordinates, including 3 base body translations (pelvis), 2 relative angles at the toes, 2 relative angles at the base of the neck, and 45 Euler angles representing the absolute orientation of the remaining bodies.

The Kalman filter is based on a discrete white noise acceleration model (DWNA) [42], in which the plant is considered as a discrete-time state-space system,

(4) $$x_{k+1}=Fx_{k}+\Gamma a_{k}$$

where $x_{k+1}$ and $x_{k}$ are the state vector at time instants k+1 and k respectively, **F** is the state propagation matrix, $a_{k}$ is the process noise vector, and Γ is the noise gain matrix. The DWNA is a second-order kinematic model, so the state vector contains the 52 degrees of freedom, q, along with their first time derivatives, $\dot{q}$. Accelerations are introduced in the system through the process noise vector **a**. This vector contains the 52 independent accelerations, being each of them a discrete-time zero-mean white sequence. Therefore, they are assumed to be constant along every time step, and their values are random variables with a zero-mean normal distribution of variance $\sigma_{a}^{2}$. This variance has dimensions of squared acceleration for the translational DOFs, and squared angular acceleration for the angular ones. In order to reduce the number of parameters, in this work the same numerical value will be used for all of them.

Taking into account that accelerations are assumed to remain constant along each time step, the state transition in Equation (4) particularized to any given DOF *i* is,

(5) $$\left[\begin{array}{c} q_{k+1}^{i}\\ {\dot{q}}_{k+1}^{i} \end{array}\right]=\left[\begin{array}{cc} 1 & \Delta t\\ 0 & 1 \end{array}\right]\left[\begin{array}{c} q_{k}^{i}\\ {\dot{q}}_{k}^{i} \end{array}\right]+\left[\begin{array}{c} \frac{1}{2}\Delta t^{2}\\ \Delta t \end{array}\right]a_{k}^{i}\;i=1,\ldots ,52$$

where Δt is the sampling period, which is fixed to 10 ms (the motion capture cameras used in this work have a maximum frame rate of 100 Hz). As can be seen in Equation (5), the state propagation and noise gain matrices defined for each DOF only depend on the time step Δt, so they are constant and equal for all of them. Therefore, matrices **F** and **Γ** for the whole system are the result of assembling these individual matrices, following the structure of the state vector **x**.

The process noise covariance matrix $Q^{i}$ has also the same form for all DOFs [42],

(6) $$Q^{i}=\left[\begin{array}{cc} \frac{1}{4}\Delta t^{4} & \frac{1}{2}\Delta t^{3}\\ \frac{1}{2}\Delta t^{3} & \Delta t^{2} \end{array}\right]\sigma_{a}^{2}$$

The **Q** matrix for the whole system is the result of assembling these individual matrices, as done for the state transition and noise gain matrices.

The observation function h(x) provides the observation vector **z**, which in this case contains the absolute *x*, *y* and *z* coordinates of the 36 optical markers, as a function of the state vector **x**,

(7) $$z_{k}=h(x_{k})+w_{k}$$

The additive term $w_{k}$ represents the noise introduced by the motion capture system, along with the skin motion artifact. Since the latter is correlated to the skeletal motion, modeling the sensor noise as a random variable following a Gaussian distribution is not strictly correct, so the Kalman filter will not be optimal. All sensors are considered independent and equally affected by noise, so the observation noise covariance matrix **R** is a diagonal matrix, whose diagonal elements are all equal to the sensor noise variance $\sigma_{s}^{2}$, which has dimensions of squared length.

**Figure 5:**
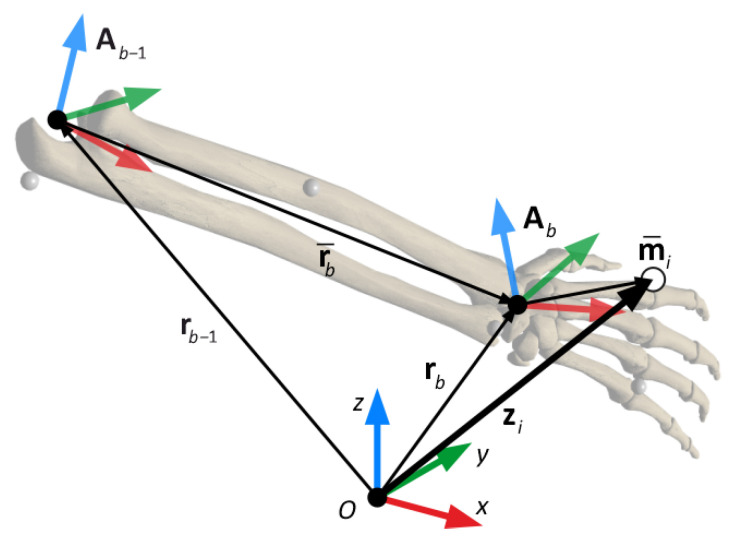
Kinematic description of the observation function h(x).

In order to compute the absolute marker positions from the system states, a recursive kinematic relationship can be established, as shown in Figure 5. The absolute position $z_{i}$ of a marker *i*, which is attached to body *b* (right hand in Figure 5), can be obtained from the following recursive relationships,

(8) $$\begin{array}{l} z_{i}=r_{b}+A_{b}{\bar{m}}_{i}\\ r_{b}=r_{b-1}+A_{b-1}{\bar{r}}_{b} \end{array}$$

where $r_{b}$ is the absolute position of the proximal joint of body *b*, $A_{b}$ is the rotation matrix of the same body, which depends on its three orientation angles, and ${\bar{m}}_{i}$ is the local position vector of marker *i* within the local frame of body *b*. In the observation model, the markers are considered as rigidly attached to the skeleton, so ${\bar{m}}_{i}$ is a constant vector. The vector $r_{b}$ itself can be obtained in a recursive way from the position vector $r_{b-1}$ and orientation matrix $A_{b-1}$ of the preceding body in the kinematic chain, knowing that ${\bar{r}}_{b}$ is the position of the proximal joint of body *b* in the local frame of *b* − 1, which, due to the rigid body assumption, is considered constant. This recursive process starts at the pelvis, whose position vector is contained directly in **q** and, consequently, in **x**.

The local position vectors ${\bar{r}}_{b}$ and ${\bar{m}}_{i}$ must be scaled prior to running the Kalman filter, in order for the model to adjust to the experimental data. This is performed by solving, at a reference pose, a nonlinear least squares optimization problem, in which the design variables are a set of scale factors **k** and the skeletal degrees of freedom **q**, being the objective function the quadratic error between measured and estimated marker positions,

(9) $$\underset{q,k}{\min}f(q,k)={\left[h_{a}(x,k)-z\right]}^{T}\left[h_{a}(x,k)-z\right]$$

where $h_{a}(x,k)$ is an augmented version of the observation function that also takes the scale factors as input variables. The resulting scale factors are then used to scale the ${\bar{r}}_{b}$ and ${\bar{m}}_{i}$ vectors that will be used in h(x). It has been found that the Levenberg-Marquardt algorithm works very well for this problem, converging in a very robust way even from rough initial estimates.

The Kalman filter algorithm follows a recursive predictor-corrector scheme. It uses the current estimate of the state vector, ${\hat{x}}_{k}$, along with the sensor measurements, $z_{k+1}$, to obtain an optimal estimate ${\hat{x}}_{k+1}$ at the next time step. In the predictor stage, the state estimate is updated by means of the state transition matrix **F**, leading to the so-called *a priori* estimate ${\hat{x}}_{k+1}^{-}$. The estimate covariance matrix **P** is updated accordingly, by using matrices **F** and **Q**,

(10) $$\begin{array}{l} {\hat{x}}_{k+1}^{-}=F{\hat{x}}_{k}\\ P_{k+1}^{-}=FP_{k}F^{T}+Q \end{array}$$

The state estimate at the first time step, ${\hat{x}}_{0}$, will contain the initial independent positions, $q_{0}$, along with the corresponding velocities, ${\dot{q}}_{0}$. The positions are obtained after solving the initial marker labeling problem, which will be described later. The initial velocities are unknown, as well as the value of $P_{0}$, so they are both set to zero, but they converge quickly to their correct values after a short transient.

The corrector stage uses the sensor measurements $z_{k+1}$ to find the optimal *a posteriori* estimate ${\hat{x}}_{k+1}$, as well as its corresponding covariance matrix, $P_{k+1}$,

(11) $$\begin{array}{l} K_{k+1}=P_{k+1}^{-}H_{k+1}^{T}{\left(H_{k+1}P_{k+1}^{-}H_{k+1}^{T}+R\right)}^{-1}\\ {\hat{x}}_{k+1}={\hat{x}}_{k+1}^{-}+K_{k+1}\left[z_{k+1}-h(x_{k+1}^{-})\right]\\ P_{k+1}=\left(I-K_{k+1}H_{k+1}\right)P_{k+1}^{-} \end{array}$$

In these equations, $H_{k+1}$ is the Jacobian matrix of the observation function, evaluated at ${\hat{x}}_{k+1}^{-}$. This matrix can be computed very efficiently due to the recursive nature of h(x). Moreover, it is quite sparse, due to the usage of absolute angles as state variables: most rotation matrices will only depend on three angles, greatly simplifying their derivatives. In addition, the gradient used in the scale optimization problem shown in Equation (9) mostly coincides with this matrix, which is very convenient from the implementation point of view.

Since this algorithm is recursive and each step can be evaluated very efficiently, it can be used for real-time motion reconstruction and visualization, as opposed to the previously mentioned methods, which provide skeletal motion after post-processing the captured data. In order to achieve on-the-fly motion reconstruction, the marker labeling and occlusion issues must be addressed.

The problem of initial marker labeling is addressed by using a simple heuristic method to identify the markers. The procedure consists of checking their relative positions at the initial time step, according to a reference pose. Then, the same Levenberg-Marquardt optimization algorithm previously used for scaling the model is used here to fit the DOFs to the measured markers. This time, the objective function uses the regular observation function h(x) with the scale factors already applied to the local position vectors, so only the positions $q_{0}$ are considered as design variables. If the objective function value after the optimization (i.e., the fitting error) is below a certain threshold, the marker order is considered valid, and the iterative process of the Kalman filter can begin.

During the execution of the Kalman filter, marker labeling must be carried out on the fly, between the predictor in Equation (10) and the corrector in Equation (11). This is because, for several reasons, the raw measurement vector $z_{k+1}^{r}$ obtained from the cameras cannot be directly used within the corrector. First, the markers are provided as an unsorted list by the cameras. Second, some markers may be missing due to occlusions. Third, other bright objects present during the motion capture can be incorrectly identified as markers. Therefore, the raw measurement $z_{k+1}^{r}$ must be correctly labeled and sorted, the missing markers need to be identified, and all spurious markers have to be discarded, in order to get the “clean” measurement vector $z_{k+1}$. After the Kalman filter predictor has computed the a priori state estimate $x_{k+1}^{-}$, the observation function h(x) is evaluated at that point to obtain the corresponding set of estimated marker positions ${\hat{z}}_{k+1}$. Ideally, these estimated markers would coincide with the measured ones $z_{k+1}^{r}$, and this fact can be used to identify the measured markers by using a simple, nearest-neighbor approach. First, a matrix of squared cross-distances **D** is built, such that

(12) $$D_{ij}={\left({\hat{z}}_{i}-z_{j}^{r}\right)}^{T}\left({\hat{z}}_{i}-z_{j}^{r}\right)$$

where ${\hat{z}}_{i}$ contains the estimated *x*, *y* and *z* coordinates of marker *i*, and $z_{j}^{r}$ is the position vector of measured marker *j*. By setting a maximum search distance, estimated markers that do not have a measured one close enough are considered as missing, and the remaining ones are assigned to their closest measured counterparts. Any marker from $z_{k+1}^{r}$ remaining unassigned, after all estimated markers have been either paired to their measured counterparts or marked as missing, are regarded as spurious, so they are discarded. In order to avoid resizing vectors and matrices at runtime, missing markers are set to zero in **z**, and the same is done to their corresponding rows in **H**, so they do not affect the correction.

The EKF can provide a smoothing effect depending on the tuning of its parameters, so in this case there is no need for filtering the marker trajectories. If the sensor noise variance $\sigma_{s}^{2}$ is fixed to a constant value, the smoothing can be controlled by the process noise variance, i.e., the acceleration variance $\sigma_{a}^{2}$. Low values of the variance limit the accelerations the system can reach at every time step, thus having a smoothing effect on the resulting position histories, while high values of the variance allow for larger accelerations, so that the system can follow the sensors (i.e., the markers) more closely, at the expense of introducing sensor noise into the reconstructed motion. In this work, the accelerations are obtained by further filtering the independent positions, and differentiating them twice to obtain velocities and accelerations. There exist higher order state-space models that include accelerations in the state vector, but they present two major issues when used with position sensors only: the resulting accelerations are noisy and delayed, and some unwanted oscillations may appear in the resulting motion for certain values of the filter parameters.

The analyst has in this case two parameters for tuning the obtained accelerations: the process noise variance and the cutoff frequency of the Butterworth filter. In order to find their optimum values, the use of accelerometers can be of great help.

#### 2.2.5. Calculation of the Accelerations

In order to make the accelerations obtained from the optical system directly comparable to those obtained by the inertial sensors, it was necessary to transform the former into the local axes of the corresponding IMUs, and to add the gravity effect to them. Such an acceleration obtained from the optical system will be called hereafter the virtual acceleration, as it comes from a virtual accelerometer. It is possible to do the opposite, i.e., to rotate the IMU measurements to the global frame of reference instead, subtracting the acceleration of gravity afterwards. However, this procedure involves mixing data from both systems (to rotate the IMU accelerations to the global frame), so the first alternative seems more appropriate.

**Figure 6:**
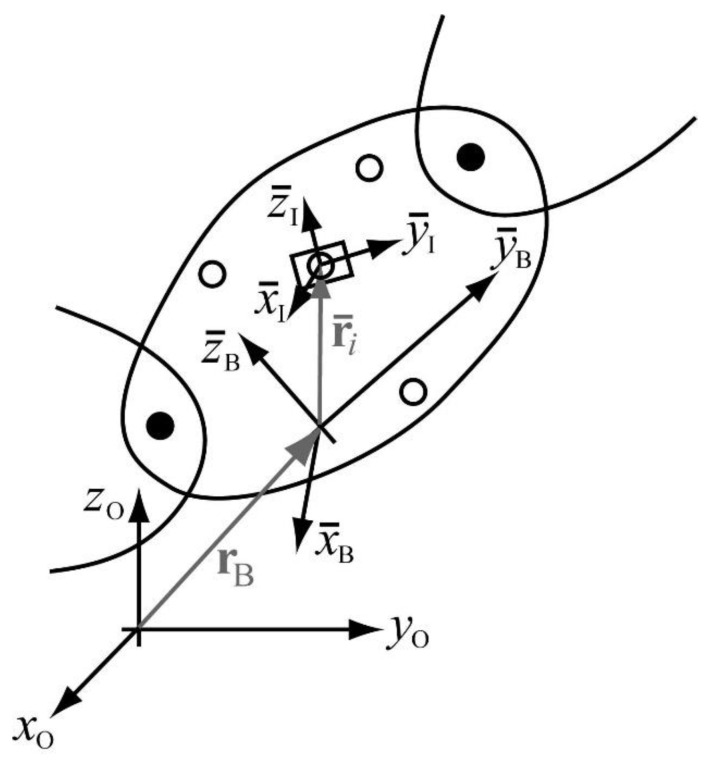
Kinematics at segment level to get the virtual acceleration (acceleration of the point of the body where the IMU is attached) from the optical motion capture recordings. The black dots represent joints connecting the segment with its neighbors. The white dots represent the markers attached to the segment.

Figure 6 shows a segment or body of the multibody model of the human skeleton, where the black dots are joints connecting the segment with its neighbors, and the white dots are the markers attached to the segment. The small rectangle represents the IMU attached to the segment, which in turn has a marker attached to it, as also shown in Figure 3. The local reference frame of the body is denoted by B, a moving frame rigidly attached to the body, and its origin is defined in frame O by the position vector $r_{B}$ while the local position of the IMU in frame B is given by the constant vector ${\bar{r}}_{i}$ (*i* is the number of the IMU attached to that particular body). The following equation can be written,

(13) $$r_{i}=r_{B}+R_{\mathrm{OB}}{\bar{r}}_{i}$$

where $r_{i}$ is the position vector of the IMU in frame O and $R_{\mathrm{OB}}$ is the rotation matrix between frames B and O. Then, ${\bar{r}}_{i}$ can be worked out as,

(14) $${\bar{r}}_{i}=R_{\mathrm{OB}}^{T}\left(r_{i}-r_{B}\right)$$

Regarding the orientations, the following relation stands,

(15) $$R_{\mathrm{OB}}{\bar{R}}_{\mathrm{BI}}=R_{\mathrm{OE}}^{i}R_{\mathrm{EI}}^{i}$$

being ${\bar{R}}_{\mathrm{BI}}$ the unknown constant rotation matrix between frames B and I. Superscript *i* in the rotation matrices of the right-hand side refers to the number of the IMU attached to that particular body. From Equation (15), the constant rotation matrix ${\bar{R}}_{\mathrm{BI}}$ can be worked out as,

(16) $${\bar{R}}_{\mathrm{BI}}=R_{\mathrm{OB}}^{T}R_{\mathrm{OE}}^{i}R_{\mathrm{EI}}^{i}$$

The two constant terms worked out in Equation (14) and Equation (16), respectively, must be determined in order to obtain, later on, the virtual acceleration. To this end, a capture of a static pose of the subject is recorded by both the optical and inertial systems. From the positions of the markers, $r_{i}$, $r_{B}$ and $R_{\mathrm{OB}}$ can be derived, so that ${\bar{r}}_{i}$ is calculated from Equation (14). On the other hand, the constant matrix $R_{\mathrm{OE}}^{i}$ had been obtained in the calibration process carried out during the preliminary test, while $R_{\mathrm{EI}}^{i}$ can be derived from the orientation provided by the IMU, so that ${\bar{R}}_{\mathrm{BI}}$ is calculated using Equation (16). It must be noted that this is the only point, along the process of getting the virtual acceleration, in which the orientation provided by the IMU is used. However, this does not induce a significant error, since the estimated orientations are much more accurate in static conditions.

Once the constant terms ${\bar{r}}_{i}$ and ${\bar{R}}_{\mathrm{BI}}$ have been determined in the described preprocess, the history of the virtual acceleration can be derived from the info recorded by the optical system. At each time point, the global acceleration of the point where the IMU is attached, expressed in frame O, can be obtained by differentiating Equation (13) twice with respect to time,

(17) $${\ddot{r}}_{i}={\ddot{r}}_{B}+{\ddot{R}}_{\mathrm{OB}}{\bar{r}}_{i}$$

where ${\ddot{r}}_{B}$ and ${\ddot{R}}_{\mathrm{OB}}$ are calculated as the second derivative with respect to time of the position data obtained from the optical motion capture. The virtual acceleration, still expressed in frame O, is obtained by including the gravity effect into the acceleration given by the Equation (17),

(18) $$a_{i}={\ddot{r}}_{i}+g$$

being **g** the gravity vector (9.81 m/s^2^ in the positive vertical direction, as it would be perceived by the IMU). To get the virtual acceleration, vector $a_{i}$ must be expressed in the local frame of the IMU, I,

(19) $${\bar{a}}_{i}=R_{\mathrm{OI}}^{T}a_{i}$$

with,

(20) $$R_{\mathrm{OI}}=R_{\mathrm{OB}}{\bar{R}}_{\mathrm{BI}}$$

where $R_{\mathrm{OB}}$ is calculated from the optical motion capture. Compacting Equations (17)–(20) into a single expression, the virtual acceleration can be written as,

(21) $${\bar{a}}_{i}={\bar{R}}_{\mathrm{BI}}^{T}R_{\mathrm{OB}}^{T}\left({\ddot{r}}_{B}+{\ddot{R}}_{\mathrm{OB}}{\bar{r}}_{i}+g\right)$$

Therefore, the acceleration directly measured by the IMU can now be compared to the virtual acceleration provided by the Equation (21) from the measurements of the optical system, and the filtering parameters applied in the latter can be adjusted so as to yield the optimal correlation. The error was measured as the root-mean-square error (RMSE) between the histories of the two accelerations compared, the results being shown in Section 3.

## 3. Results

### 3.1. Preliminary Test and Calibration

**Figure 7:**
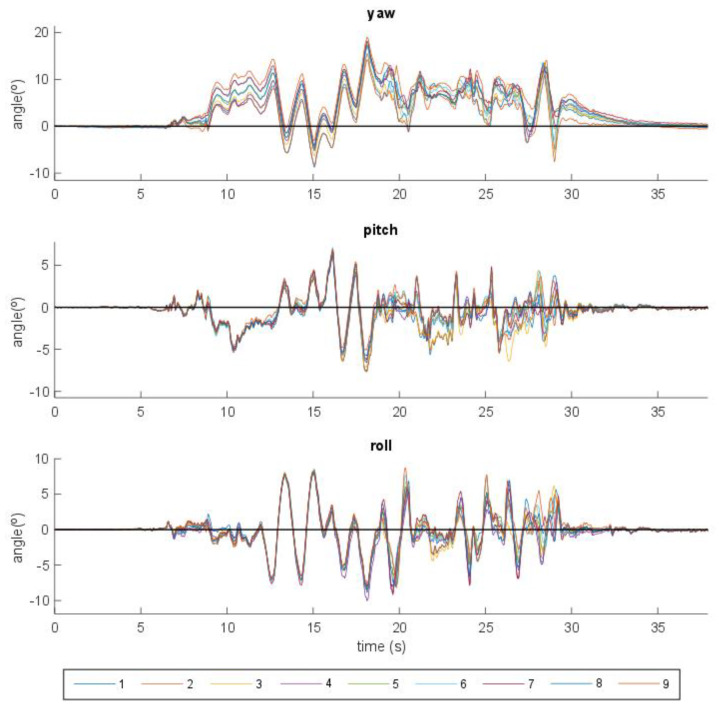
Orientation errors in roll, pitch and yaw incurred by the nine IMUs with respect to the optical system (reference).

As explained in Section 2, the orientation error committed by the *i*th IMU, *i* = 1,…,9, at each time point, was obtained by calculating the roll, pitch and yaw angles of $R_{\mathrm{OI}}^{i}$, and comparing them with the roll, pitch and yaw angles of $R_{\mathrm{OI}}$, taken as reference. Figure 7 shows the error incurred by each IMU in roll, pitch and yaw angles, along the time of the calibration experiment. Maximum errors of 19° in yaw (around the vertical axis) with respect to the reference (optical system) were found, while mean error differences of up to 4° were detected among IMUs. Similar results were obtained for $R_{\mathrm{OE}}^{i}$, the rotation matrix between frames E and O for the inertial sensor *i*: differences of up to 8° in yaw were detected among IMUs.

**Figure 8:**
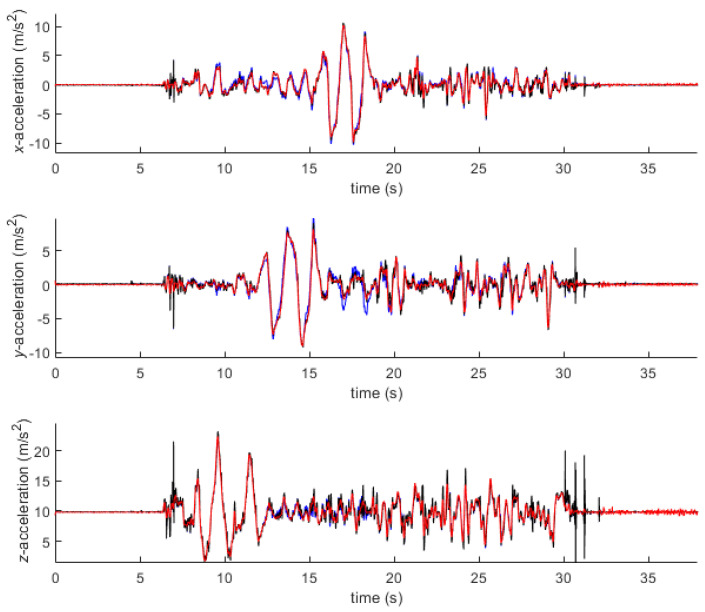
Comparison of the global acceleration of point 4 of the wooden plate, obtained by three methods: from the optical system (red); from the inertial system with the orientation provided by the inertial system (blue); from the inertial system with the orientation provided by the optical system (black).

As explained in the last paragraph of Section 2.1.2, the acceleration of point 4 of the wooden plate was obtained in three different ways: (i) from the optical system, using the marker attached to point 4; (ii) from the inertial system, using the orientation provided by the inertial system; (iii) from the inertial system, using the orientation provided by the optical system. A forward-backward 2nd order Butterworth filter with a cutoff frequency of 8 Hz was applied to the optically captured trajectories of the markers, while no filtering was applied to the inertial measurements. Figure 8 gathers the global components, expressed in frame O, of the three accelerations. While the *x*- and *z*-components are similar, significant discrepancies are observed between 15 and 20 s for the *y*-component, with a maximum error of 1.9 m/s^2^ when using the orientation provided by the IMU. Moreover, the accelerometer shows some peaks that are not captured by the optical system, for instance when the plate touches the ground after the 30 s mark. Due to the low sampling rate of the optical system, it cannot capture high-frequency events such as impacts, regardless of the filter cutoff frequency. 

### 3.2. Gait Analysis

This Section is devoted to gather the results obtained when comparing the accelerations provided by each of the seven IMUs during the gait analysis described in Section 2.2, with the so-called virtual accelerations obtained from the optical motion capture. As explained in the mentioned Section, the trajectories of the markers recorded by the optical system should be processed by a motion reconstruction method, which includes filtering of the recorded data. Therefore, the results from each of the two reconstruction methods proposed in Section 2.2 are shown in what follows.

#### 3.2.1. Vaughan’s Method

**Figure 9:**
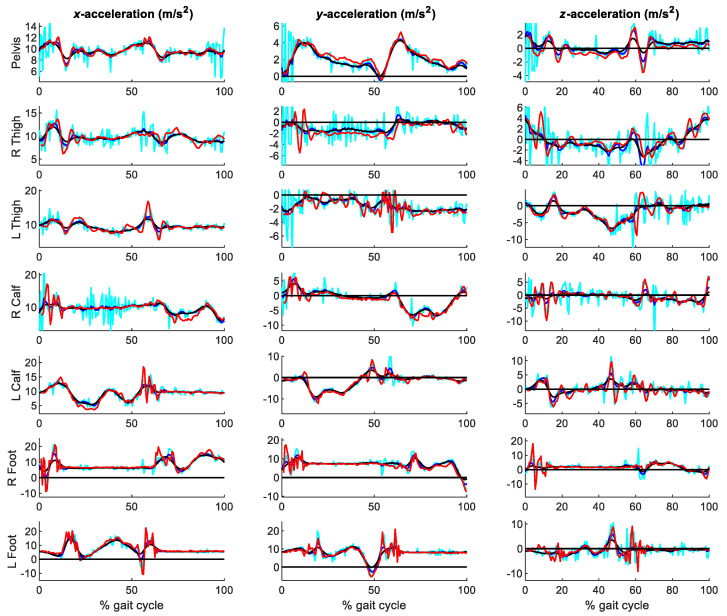
Accelerations obtained from the optical system with Vaughan’s method, for cutoff frequencies of 6 Hz (black), 12 Hz (blue) and 40 Hz (cyan), respectively, vs. accelerations measured by the IMUs (red).

**Figure 10:**
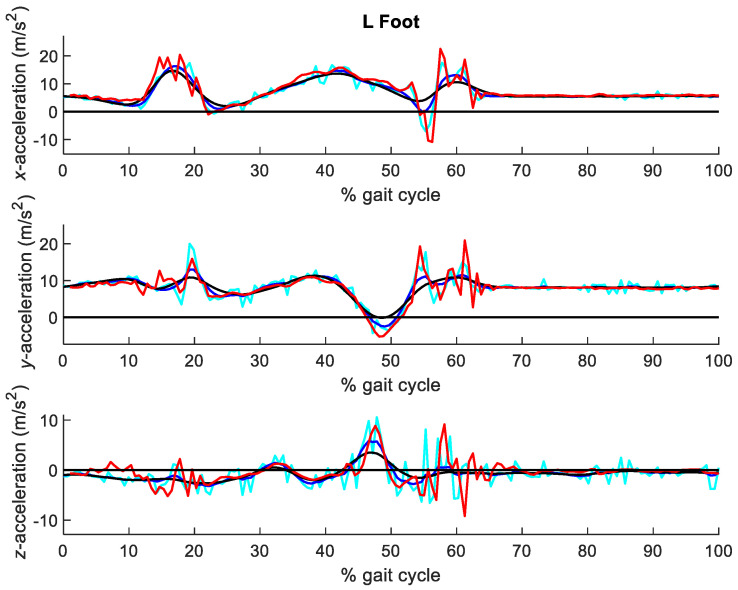
Detail of accelerations at the left foot obtained from the optical system with Vaughan’s method, for cutoff frequencies of 6 Hz (black), 12 Hz (blue) and 40 Hz (cyan), respectively, vs. accelerations measured by the IMUs (red).

The virtual accelerations obtained after the application of the forward-backward 2nd order Butterworth filter with different cutoff frequencies were compared with those directly measured by the IMUs. Figure 9 shows the three components of the accelerations at the seven segments analyzed for cutoff frequencies of 6, 12 and 40 Hz, while Figure 10 provides more detail for the left foot. Table 1 gathers the RMSE of the optical-system based accelerations, with cutoff frequencies ranging between 6 and 40 Hz, with respect to those directly measured by the inertial system.

It can be seen that the influence of the filtering parameter is significant. For high cutoff frequencies (above 20 Hz), the accelerations were too noisy, with peak errors over 5 m/s^2^. Conversely, for low cutoff frequencies (below 8 Hz), the accelerations were too smooth, not reaching the experimental peak measurements of the inertial sensors. As opposed to the preliminary test, some acceleration peaks can be captured by the optical system at high cutoff frequencies, due to the softer contacting materials involved in this case, but at the cost of very noisy accelerations along the whole capture. The lowest errors were obtained for a cutoff frequency of 12 Hz, as highlighted in Table 1.

#### 3.2.2. Extended Kalman Filter

The accelerations obtained after the application of different values of the process noise standard deviation $\sigma_{a}$ (with the sensors noise standard deviation $\sigma_{s}$ fixed to 0.001 m), along with the application of the forward-backward 2nd order Butterworth filter with different cutoff frequencies to the position data, were compared to those directly measured by the IMUs. The units for $\sigma_{a}$ are omitted in what follows for the sake of brevity, since they depend on the associated degree of freedom: for translational DOFs, $\sigma_{a}$ is expressed in m/s^2^ whereas for rotational DOFs it is in rad/s^2^.

**Figure 11:**
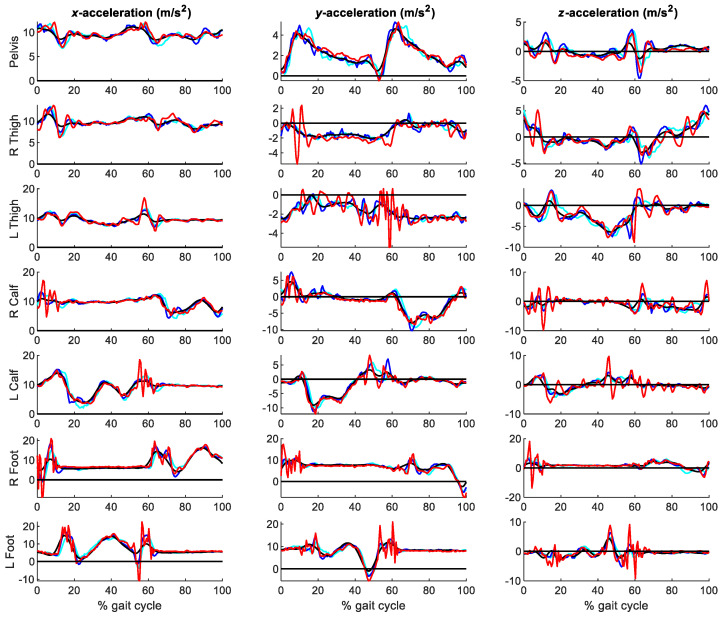
Accelerations obtained from the optical system with the EKF for combined process noise variances and cutoff frequencies of 0.1/30 Hz (black), 1/20 Hz (blue) and 50/6 Hz (cyan), respectively, vs. accelerations measured by the IMUs (red).

**Figure 12:**
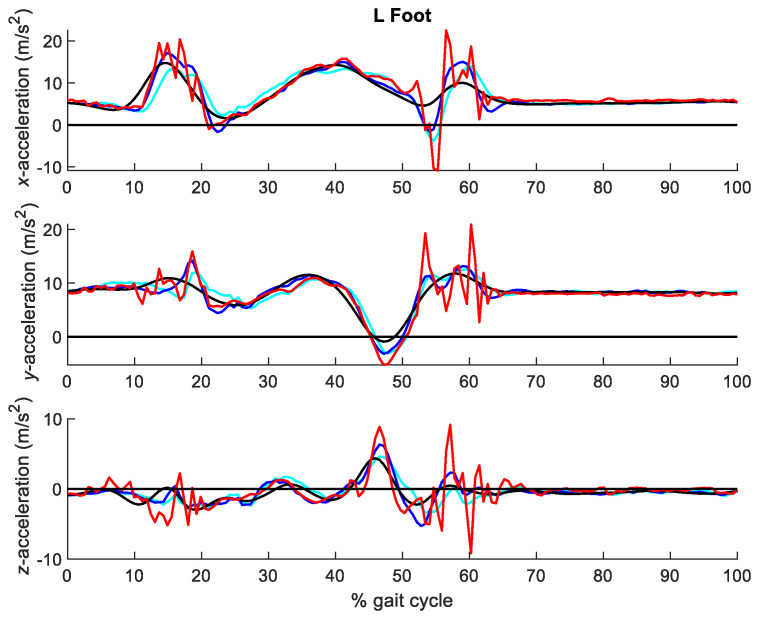
Detail of accelerations at the left foot obtained from the optical system with the EKF-based method for combined process noise standard deviations and cutoff frequencies of 0.1/20 Hz (black), 1/15 Hz (blue) and 50/6 Hz (cyan), respectively, vs. accelerations measured by the IMUs (red).

Figure 11 shows the three components of the accelerations at the seven segments analyzed for combinations of $\sigma_{a}$ and cutoff frequencies of 0.1/30 Hz, 1/20 Hz and 50/6 Hz, while Figure 12 provides more detail for the left foot. Table 2 gathers the RMSE of the optical-system based accelerations with $\sigma_{a}$ ranging between 0.1 and 50 m/s^2^ (or rad/s^2^, depending on the corresponding coordinate), and cutoff frequencies ranging between 6 and 30 Hz, with respect to those directly measured by the inertial system. 

It can be seen in Figure 11 and Figure 12 that the accelerations obtained with the EKF were smoother than those obtained with Vaughan’s method, and that the experimental peak measurements of the inertial sensors were better dissociated from the noise peaks. Moreover, Table 2 presents lower values of the RMSEs. The best results were obtained for a process noise standard deviation of 1 m/s^2^ combined with a 20 Hz Butterworth filter.

In addition to obtaining better accelerations, it should be noted that the EKF automatically ensures kinematic consistency, whereas Vaughan’s method shows joint distance variations above 1 cm along the gait cycle, so the resulting motion would require further post-processing depending on the intended application.

## 4. Discussion and Limitations of the Study

This work proposes both an extended Kalman filter that facilitates optical motion capture, and an objective filter-tuning procedure that improves the resulting accelerations in gait analysis by using accelerometer data. First, a preliminary test including nine IMUs was carried out to assess the errors incurred by the inertial sensors in the measured orientations and accelerations. Second, the gait analysis of a healthy subject was performed. Both optical motion capture and inertial motion capture (using the seven most accurate IMUs out of the nine tested in the spot check) were recorded. The motion was then reconstructed by the classic Vaughan’s method (filtering the marker trajectories with a Butterworth filter) and by the proposed EKF (applying a process noise variance and filtering the marker trajectories with a Butterworth filter), and the accelerations measured by the IMUs were used to tune the parameters of the filters for both methods.

As observed earlier in [11,12,13], the preliminary test highlighted the IMUs limitation to yield an accurate orientation. These errors depend on the calibration of the accelerometers and magnetometers, and on the algorithm used to estimate the orientations. Brodie [13] showed that it is possible to reduce the errors by substituting the commercial algorithm implemented in the inertial sensors by an improved one, which is consistent with the experience of the authors using other algorithms [35,36], but even in this case their accuracy remains limited. Therefore, although the performance of the IMUs has been improved in the last decade, optoelectronic systems are still used as the golden standard reference [18,19,43]. For this reason, it was decided to reduce the use of the orientations provided by the IMUs to a minimum for the gait analysis, taking as reference the local accelerations measured by the IMUs, and applying all the required transformations in the optical methods so as to obtain the accelerations, denoted as virtual accelerations, which are directly comparable to those provided by the inertial sensors.

This decision was enforced after observing, in the preliminary test, the effect of the orientation errors incurred by the IMUs on the global accelerations. As reported by Woodman [44], it is necessary to have very accurate rotation sensors in inertial navigation systems because the precise orientation of the body must be known in order to mathematically calculate the gravitational acceleration to find the translational acceleration. As observed in Figure 8, the gravitational acceleration was incorrectly estimated and appeared as translational acceleration perpendicular to the gravitational vector. To alleviate this problem, the orientations obtained from the optical system could be used instead but, as they are sensitive to the filter tuning, the resulting global accelerations from the IMUs would be distorted too.

The virtual accelerations obtained by Vaughan’s method were very sensitive to the filtering applied to the trajectories of the markers. Best matches with experimental values were observed for cutoff frequencies ranging between 10 and 15 Hz. Bartlett [45] stated that cutoff frequencies between 4 and 8 Hz are often used in filtering movement data, while the OpenSim software [40] recommends to use a cutoff frequency of 6 Hz. However, it was observed that by using low cutoff frequencies, the accelerations were too smooth and the peaks measured by the IMUs were not reached. Schreven et al. [31] found that filtering the data with a cutoff frequency of 6 Hz decreases the accuracy of the reconstructed kinematics and, hence, can affect the accuracy of the joint moments obtained from inverse dynamics, as shown in [46].

Regarding the EKF method, apart from its robustness and simplicity of use, it showed a better accuracy in the resulting accelerations. The best filtering was obtained for a plant noise variance of 1 m/s^2^ (or rad/s^2^, depending on the corresponding coordinate) and a cutoff frequency of 20 Hz. Noise was eliminated, peaks measured by the IMUs were almost reached, and the resulting RMSEs were better than those incurred by Vaughan’s method. Moreover, the EKF offered consistent kinematics by providing constant lengths of the body segments along the motion. Vaughan’s method is similar to those proposed in [19] and, like them, does not impose the kinematic constraints to compute the joint kinematics from the marker trajectories. Therefore, it would require an additional step to correct these inconsistencies before dynamic analysis.

Although gait may be perceived as a smooth activity, acceleration peaks due to foot impact are observed in Figure 10 and Figure 12, captured by the inertial system. In fact, they were also captured by the optical system when sampling at 100 Hz. Focusing on the acceleration peaks due to left foot landing, happening at around 60% of the gait cycle, it can be seen in Figure 10 that filtering with a cutoff frequency of 40 Hz already allows to capture them, but at the cost of keeping a lot of noise in the rest of the signal. On the other hand, using a cutoff frequency under 30 Hz provides a much cleaner signal, but notably oversmooths the impact peaks. Therefore, the procedure proposed in this paper can be useful for other researchers to evaluate existing filtering methods, design new ones and chose the best filtering parameters, but also to select the best capture frequency for their applications, because they will be able to distinguish between peaks due to noise and peaks due to actual motion.

The conducted study has been based on the results obtained from the gait analysis of one single subject. Although it could be expected that the frequency content of the motion signals is more dependent on the type of activity than on the particular subject performing it, tests including a greater number of subjects would be advisable in order to confirm the presented conclusions. This has been the reason to include the word ‘preliminary’ in the title.

## 5. Conclusions

The conclusion is twofold. First, when performing motion capture and analysis using a marker-based optical system, the extended Kalman filter significantly streamlines the motion capture and reconstruction process, since it facilitates automatic marker labeling, and manages occlusions and reflections in a robust and efficient way. Second, the availability of accelerations measured by inertial sensors can be very helpful for the tuning of the filters, no matter which motion reconstruction method is used. Consequently, the reliability of the obtained accelerations is improved.

## Figures and Tables

**Table 1:** RMSE of the accelerations obtained from the optical system through Vaughan’s method with different cutoff frequencies, with respect to the accelerations measured by the IMUs, taken as reference. The row with the lowest mean RMSE is highlighted in red.

Cutoff Freq. (Hz)	RMSE (m/s^2^)							
	Pelvis	R Thigh	L Thigh	R Tibia	L Tibia	R Foot	L Foot	Mean
6	0.626	1.034	1.123	1.583	1.502	2.743	2.485	1.585
8	0.578	1.005	1.071	1.538	1.448	2.640	2.405	1.336
10	0.559	0.997	1.043	1.515	1.428	2.571	2.362	1.309
12	0.559	1.003	1.031	1.508	1.426	2.529	2.339	1.299
15	0.583	1.034	1.036	1.521	1.445	2.504	2.328	1.306
20	0.680	1.138	1.086	1.607	1.510	2.526	2.354	1.363
25	0.840	1.303	1.181	1.771	1.602	2.590	2.422	1.464
30	1.045	1.517	1.314	2.002	1.706	2.679	2.526	1.599
40	1.531	2.035	1.654	2.602	1.931	2.897	2.815	1.933

**Table 2:** RMSE of the accelerations obtained from the optical system through the EKF-based method with different combinations of process noise standard deviations and cutoff frequencies, with respect to the accelerations measured by the IMUs, taken as reference. The row with the lowest mean RMSE is highlighted in red.

Acc. Std. (m/s^2^ or rad/s^2^)	Cutoff Freq. (Hz)	RMSE (m/s^2^)							
		Pelvis	R Thigh	L Thigh	R Tibia	L Tibia	R Foot	L Foot	Mean
0.1	6	0.717	1.047	1.195	1.663	1.624	2.649	2.430	1.618
0.1	10	0.679	1.020	1.155	1.679	1.618	2.530	2.338	1.377
0.1	15	0.676	1.018	1.136	1.678	1.614	2.433	2.274	1.354
0.1	20	0.682	1.022	1.133	1.674	1.611	2.377	2.229	1.341
0.1	25	0.690	1.028	1.135	1.672	1.612	2.341	2.198	1.335
0.1	30	0.698	1.035	1.140	1.672	1.615	2.318	2.176	1.332
0.5	6	0.606	1.011	1.108	1.513	1.442	2.553	2.274	1.313
0.5	10	0.545	0.969	1.030	1.514	1.412	2.340	2.105	1.239
0.5	15	0.557	0.965	1.017	1.524	1.415	2.183	2.004	1.208
0.5	20	0.582	0.977	1.034	1.530	1.424	2.098	1.943	1.198
0.5	25	0.608	0.996	1.058	1.538	1.439	2.049	1.907	1.199
0.5	30	0.634	1.018	1.083	1.553	1.458	2.018	1.887	1.207
1	6	0.604	1.006	1.099	1.484	1.433	2.565	2.267	1.307
1	10	0.538	0.963	1.026	1.454	1.383	2.330	2.072	1.221
1	15	0.562	0.961	1.033	1.457	1.379	2.161	1.955	1.188
1	20	0.601	0.978	1.067	1.466	1.393	2.074	1.890	1.183
1	25	0.639	1.004	1.105	1.483	1.417	2.026	1.857	1.191
1	30	0.679	1.036	1.143	1.511	1.446	1.999	1.843	1.207
10	6	0.632	0.987	1.132	1.473	1.439	2.580	2.324	1.321
10	10	0.576	0.926	1.086	1.393	1.343	2.344	2.131	1.225
10	15	0.606	0.928	1.115	1.374	1.300	2.174	2.002	1.187
10	20	0.665	0.973	1.168	1.397	1.307	2.084	1.934	1.191
10	25	0.739	1.041	1.228	1.452	1.342	2.037	1.909	1.218
10	30	0.823	1.124	1.292	1.538	1.391	2.014	1.913	1.262
50	6	0.633	0.989	1.134	1.478	1.442	2.578	2.330	1.323
50	10	0.574	0.933	1.088	1.401	1.348	2.343	2.142	1.229
50	15	0.603	0.943	1.119	1.386	1.307	2.177	2.018	1.194
50	20	0.666	1.000	1.179	1.424	1.319	2.091	1.955	1.204
50	25	0.753	1.085	1.253	1.507	1.359	2.047	1.937	1.243
50	30	0.859	1.190	1.336	1.632	1.416	2.032	1.953	1.302

## Data Availability

The data presented in this study are available on request from the corresponding author. The data are not publicly available due to privacy restrictions.
